# A New Method for Extracting Refined Sketches of Ancient Murals

**DOI:** 10.3390/s24072213

**Published:** 2024-03-29

**Authors:** Zhiji Yu, Shuqiang Lyu, Miaole Hou, Yutong Sun, Lihong Li

**Affiliations:** 1 School of Geomatics and Urban Spatial Informatics, Beijing University of Civil Engineering and Architecture, Beijing 102616, China; 2108160222005@stu.bucea.edu.cn (Z.Y.); lvshuqiang@bucea.edu.cn (S.L.); 210813j121019@stu.bucea.edu.cn (Y.S.); 2 Beijing Key Laboratory for Architectural Heritage Fine Reconstruction & Health Monitoring, Beijing 102616, China; 3 Yungang Research Institute, Datong 037007, China; 13233616482@163.com

**Keywords:** digital conservation of murals, image enhancement, edge detection, sketch extraction

## Abstract

Mural paintings, as the main components of painted cultural relics, have essential research value and historical significance. Due to their age, murals are easily damaged. Obtaining intact sketches is the first step in the conservation and restoration of murals. However, sketch extraction often suffers from problems such as loss of details, too thick lines, or noise interference. To overcome these problems, a mural sketch extraction method based on image enhancement and edge detection is proposed. The experiments utilize Contrast Limited Adaptive Histogram Equalization (CLAHE) and bilateral filtering to enhance the mural images. This can enhance the edge features while suppressing the noise generated by over-enhancement. Finally, we extract the refined sketch of the mural using the Laplacian Edge with fine noise remover (FNR). The experimental results show that this method is superior to other methods in terms of visual effect and related indexes, and it can extract the complex line regions of the mural.

## 1. Introduction

Murals are the treasures of human civilization, with high artistic value and historical significance. However, due to environmental changes and human activities, many murals have suffered various degrees of damage. Extracting sketches is crucial for conserving and restoring these painted cultural relics. The color and texture information of murals are complex and diverse, and they are often based on sketches that use techniques like thick line variation and an interplay of light and dark to express different artistic styles. Therefore, accurately locating the edges in murals is crucial for extracting their fine contours. Traditionally, sketches are manually copied by painters. This method is complicated, time-consuming, and often lacks accuracy. To improve the accuracy of sketch extraction, researchers have explored many automatic methods to extract mural sketches.

The current automatic sketch extraction methods are mainly based on edge detection methods, which can be divided into two categories: traditional edge detection algorithms and deep learning edge detection methods. Traditional edge detection methods mainly obtain edges by using the base features of the image (color, luminance, gradient), and such methods locate the edges of an image by identifying the locations in the image where the pixel features change drastically. Lawrence Roberts proposed the first first-order edge detection algorithm, Robert, to obtain the difference between two neighboring pixels in the diagonal direction. On the other hand, second-order differentiation of the image was performed to form local extreme points near its resulting zeros and to detect the zeros by setting a threshold, such as Canny and so on [1]. Many sketch extraction methods have been derived from these algorithms. Sun et al. [2] have designed an automatic sketch generation system based on the FDoG algorithm. Zitnick et al. [3] have proposed a novel method for generating object bounding box proposals using edges. P. Arbelaez et al. [4] have proposed an artificial feature design method based on information theory (gPb-owt-ucm algorithm). These methods can effectively extract edge details in images. However, they are too sensitive to regions with dense variations such that they can easily extract more noise while the extracted sketch is not clear enough.

With the development of deep learning techniques, many edge detection methods based on deep learning have emerged. These methods take advantage of their models’ powerful feature extraction and learning capabilities. They can derive the edge profile of an image based on global features and contextual information. The technology is applied in many different fields. Shen et al. [5] have proposed the deep contour method, an edge detection method that employs structured forests as edge and non-edge classifiers. He et al. [6] have proposed a Bi-directional cascade network (BDCN) and used a scale enhancement module to enrich edge features. Xie et al. [7] have proposed a network, (Holistically Nested Edge Detection) HED, which mainly solved the problems of whole image training and multi-scale learning in edge detection. Pan et al. [8] have proposed a deep learning network that performs mural-to-sketch prediction by combining meaningful convolutional features in a holistic manner. Liu et al. [9] have proposed Richer convolutional features for edge detection (RCF). Hu et al. [10] have proposed an accurate edge detector using a Distance Field-Based Convolutional Neural Network (DF-CNN) to accurate end-to-end object edge detection. Zhang et al. [11] have used a combination of BDCN and U-Net to obtain edge images with less noise. These methods can extract the edge contours of murals well and do not produce broken lines easily. Samma et al. [12] have used a hybrid optimization-based model to handle the problem of face sketch recognition. Hallman et al. [13] have proposed a simple, efficient model for learning boundary detection based on a random forest classifier. Cao et al. [14] have proposed a full-scale identity supervision method, which adapts the perceptual appearance of the latent image to the target image by adversarial learning and preserves the best perceptual appearance and more distinct details. Bertasius et al. [15] have proposed an end-to-end network architecture deep edge, which combines the local and global information of images to significantly improve edge detection accuracy. Poma et al. [16] have proposed a deep learning-based edge detector, inspired by both the HED (Holistically Nested Edge Detection) and Xception networks. Pu et al. [17] have proposed a novel neural network solution, RINDNet, to jointly detect all four types of edges. Kelm et al. [18] have prioritized effective utilization of the high-level abstraction capability of a ResNet, which has led to state-of-the-art results for edge detection. Wang et al. [19] have proposed a gradient-guided dual-branch generative adversarial network for high-quality relic sketch generation. Yan et al. [20] have used an adversarial architecture to capture the differences between the two domains and have adopted identity recognition loss to preserve the detailed identifiable information of photo-sketch synthesis. Zhou et al. [21] have proposed an unsupervised learning framework that adopts a gradient-based method to generate scale-dependent pseudo-edge maps. Liu et al. [22] have used a fully convolutional neural network to generate category-agnostic edges and identify high-level semantics. Sun et al. [23] have achieved the edge detection of flames by using the convolutional neural network based on VGG16. Conversely, CNN-based methods prioritize global features over detailed information during the deep learning process, often leading to overly thick lines and a loss of detailed information. This is not favorable for extracting the sketches of murals. At the same time, mural data are difficult to obtain, which poses a challenge in obtaining sufficient training datasets and is a crucial problem that most deep learning methods struggle with. Additionally, some deep learning methods emphasize secondary refinement and the generation of extracted features rather than concentrating on the data’s original features. These factors could result in discrepancies between the extraction results and the original sketches of the murals.

Accordingly, we propose a method to extract a refined sketch of murals, which consists of two main steps: image information enhancement and edge detection. In the enhancement step, we focus on highlighting the edge information of the image. First, we use Contrast Limited Adaptive Histogram Equalization (CLAHE) to enhance the image’s contrast. Then, bilateral filtering is applied to remove the noise generated by over-enhancement. This enhanced image serves as the input for the subsequent edge detection step. In the edge detection step, we utilize the Laplacian Edge (Kornia) method with fine noise remover (FNR) for mural edge detection. The final result is a refined and accurate sketch of the mural.

Unlike previous sketch extraction methods, this method focuses more on highlighting the edge information of the original image and then extracting the edges using the edge detection method. The proposed method will extract refined, accurate, and clear sketches. The contributions of this paper can be summarized as follows:

- We design a combined method for mural image edge information enhancement, which can effectively highlight the edges in an image and suppress noise generation, solving the problem of over-enhancement for further sketch extraction.
- We combine an edge detection method with our FNR to make it more suitable for acquiring refined sketches of murals. The method detects more complex details in the murals and further filters out fine noise from the generated sketch to produce a straightforward sketch.

The remainder of the paper is organized as follows: Section 2 describes the relevant data and apparatus. Section 3 introduces the method proposed in this paper. All experimental results and analyses are shown in Section 4. A summary and some closing remarks are made in Section 5.

## 2. Experimental Data and Apparatus

### 2.1. Experimental Data

**Figure 1:**
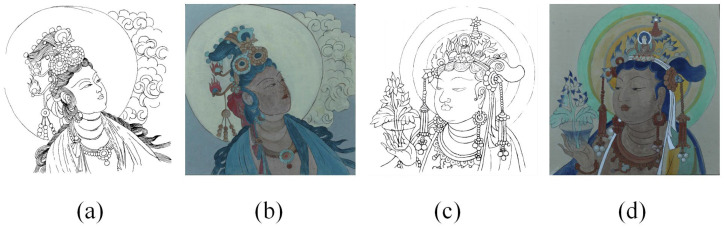
Simulated mural and its corresponding original sketch. (**a**,**b**) The original sketch and image of “SHUIYUE”, (**c**,**d**) the original sketch and image of “DAFO”, respectively.

Due to the unique characteristics of mural data, using actual murals for sketch extraction often presents challenges in obtaining ground truth. Therefore, we have adopted simulated murals as experimental data. We have produced simulated mural samples using the actual mural production process in the laboratory. We have created two murals—“SHUIYUE” and “DAFO”. First, we completed the sketches of the mural paintings and captured orthographic maps of these sketches to serve as ground truth. Then, based on these sketches, we outlined the mural lines on clay plates. Finally, we completed the painting with mural pigments, as shown in Figure 1.

### 2.2. Apparatus

Our experimental data were collected by using a D850 digital camera of the Nikon D series from Nikon Corporation, which was used to collect data on two pairs of murals with different styles. The highest resolution of the digital images captured was 8256 × 5504, with a maximum pixel of 46 M pixels and an effective pixel of 45 M pixels. When shooting, the central optical axis of the camera was perpendicular to the surface of the murals, and the camera was about 1.2 m away from the murals.

## 3. The Proposed Method

**Figure 2:**
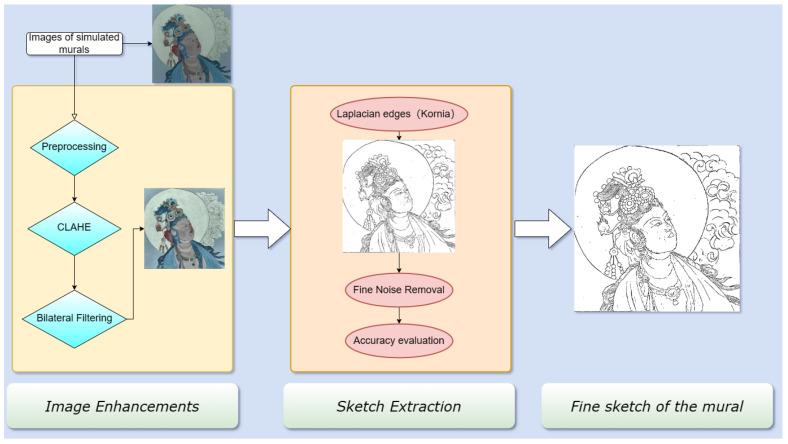
The proposed approach.

The framework proposed in this paper is shown in Figure 2. The original data of the simulated mural were captured using a digital camera. Following color transformation as a pre-processing step, we enhanced the image’s contrast using CLAHE. The over-amplified noise of the image was removed by using bilateral filtering. Finally, we used the Laplacian Edge (Kornia) with FNR to detect the image’s edges, thus obtaining a refined mural sketch.

### 3.1. Image Enhancements

#### 3.1.1. CLAHE

CLAHE is based on Adaptive Histogram Equalization (AHE) to limit the image contrast. By setting a threshold on the local region of the image, the part beyond the threshold will be evenly distributed to each part of the histogram to achieve the effect of limiting the excessive enhancement of local contrast and also, to a certain extent, to suppress noise and avoid the block effect. The core process of the algorithm is as follows:

(1) Divide the image into M × N (usually set to 8 × 8) non-overlapping and contiguous sub-regions.
(2) Calculate the average pixels of each sub-region $\bar{n}=\frac{n_{x}n_{y}}{n_{gray}}$: $n_{x}$ is the number of pixels in the *x*-axis direction of the image; $n_{y}$ is the number of pixels in the *y*-axis direction of the image; and $n_{gray}$ is the number of grey levels in the subregion.
(3) Redistribute the pixels in each subregion by gathering all of the intercepted pixels and redistributing them to various histogram grey levels by intercepting the percentage of pixel values greater than $\beta \bar{n}$; β is a controllable parameter.
(4) Apply AHE processing to the cropped area. Next, use the pixel value at the center of the equalized sub-block as a reference point. Finally, utilize bilinear interpolation to determine and output the grey value of each picture point.

**Figure 3:**
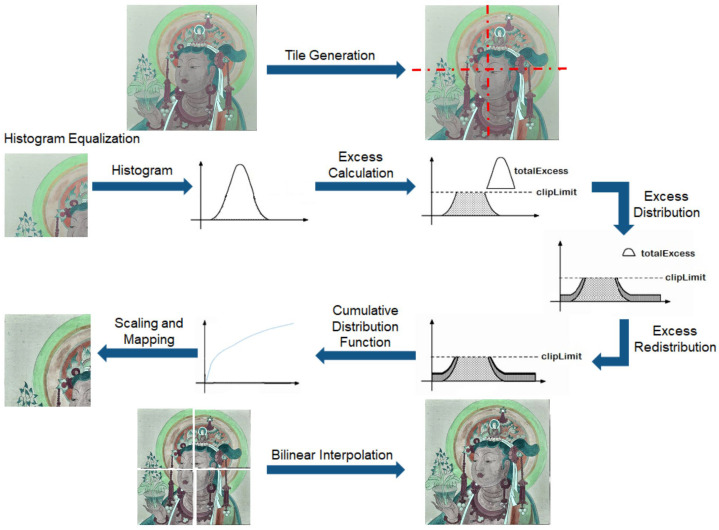
The process of CLAHE on murals.

We use “DAFO” as an example. The specific process is shown in Figure 3.

In simple terms, CLAHE is the block histogram equalization of images under certain constraints. Histogram equalization (HE) is the adjustment of the distribution (probability density) of a histogram to a uniform distribution. By applying it to an image, the histogram distribution of an image can be turned into an approximately uniform distribution using a cumulative distribution function. This magnifies the difference in pixel values at different pixel points on an image, making the image clear and organized. In this paper, the processing is completed directly with RGB images. CLAHE is performed on the R, G, and B channels, respectively. Then, the three channels are re-synthesized into one RGB image. Due to the many pigment overlays in the murals, the boundaries between different color fields tend to become blurred. Using CLAHE on murals can make this border more straightforward and improve contrast. The essence of edge detection is to detect those pixel points with abrupt changes in grey values. After contrast enhancement, the edge pixel points of the image are more easily detected.

#### 3.1.2. Bilateral Filtering

The basic idea of bilateral filtering is to consider both the domain and range of the pixels to be filtered. The weight function of bilateral filtering results from the product of the spatial proximity factor and the grey similarity factor. After introducing the weight function, the weighting result of the window pixel points is jointly determined by the spatial proximity and the grey similarity. Suppose I is a noisy image, $\hat{I}$ is a filtered image, $M\left(x,y\right)$ denotes the set of $\left(2N+1\right)\times \left(2N+1\right)$ spatial domains center on pixel $\left(x,y\right)$, and $I\left(x,y\right)$ denotes the pixel values of $\left(i,j\right)$ in $M\left(x,y\right)$. The expression of the weight coefficients of the bilateral filter is as follows:

(1) $$\;W\left(i,j\right)=\frac{W_{x}\left(i,j\right)W_{y}\left(i,j\right)}{c}$$

where $W_{x}\left(i,j\right)=e^{-\frac{{\left|i-x\right|}^{2}+{\left|i-y\right|}^{2}}{2{\sigma_{x}}^{2}}}$ is the spatial weight, $W_{y}\left(i,j\right)=e^{-\frac{{\left|I\left(i,j\right)-I\left(x,y\right)\right|}^{2}}{2{\sigma_{y}}^{2}}}$ is the range weight, and c is a constant; then, the expression for Bilateral filtering is as follows:

(2) $$\hat{I}\left(x,y\right)=\frac{\sum_{\left(i,j\right)\in M_{x,y}}W_{x}\left(i,j\right)W_{y}\left(i,j\right)I\left(i,j\right)}{\sum_{\left(i,j\right)\in M_{x,y}}W_{x}\left(i,j\right)W_{y}\left(i,j\right)}$$

**Figure 4:**
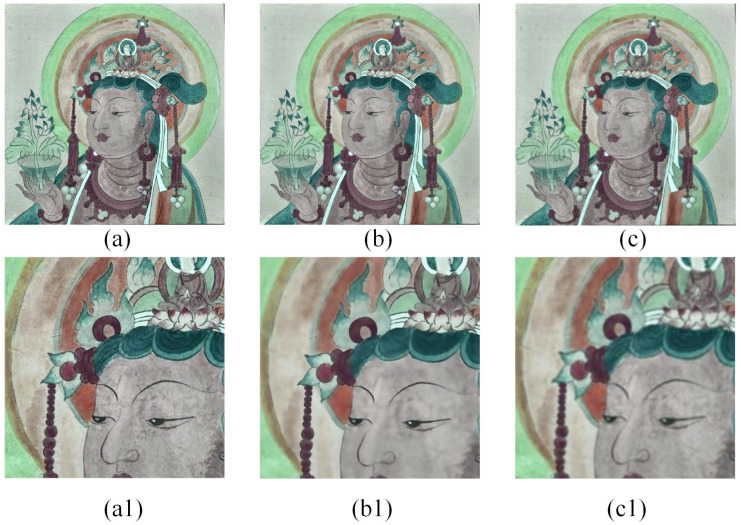
Comparison of results for different filters. (**a**) The original image (CLAHE), (**b**) the result of (**a**) using Bilateral filter, and (**c**) the result of (**a**) using Gaussian filter. The figures below (**a1**–**c1**) are the corresponding partially enlarged details.

According to Equation (1), it can be seen that the weight coefficients are affected by both the spatial proximity factor and the grey similarity factor. The spatial proximity factor increases with the decrease in the spatial position of the neighboring pixel point and the central pixel point, and the grey similarity factor increases with the reduction in the grey difference between two pixels. In the flat region, each pixel point in the filter has a similar $W_{y}\left(i,j\right)$, and$\;W_{x}\left(i,j\right)\;$plays a significant role in the filtering process. In the edge region, $W_{y}\left(i,j\right)$ on the same side of the edge is similar and much more significant than $W_{y}\left(i,j\right)$ on the other side of the edge, and $W_{y}\left(i,j\right)$ plays a significant role in the filtering process. Currently, the weights of the pixel points on the other side have almost no effect on the filtering result, and the edge information is protected. A certain degree of adaptivity is shown. When a noise point occurs in a flat area, the signals around the noise point have small weights. After normalization, these weights are boosted, hence there is a filtering effect on the noise points as well. Unlike classical Gaussian filtering, bilateral filtering combines pixel grey scale similarity with spatial information. This approach not only retains the rich details of the image but also suppresses coherent spots in homogeneous areas, as shown in Figure 4. The mural image after CLAHE produces cluttered information resulting from over-enhancement. Bilateral filtering can smooth out this unwanted information while keeping the image edges and maintaining detail texture regions. This is more conducive to extracting sketches of murals. Details of the sketch extraction will be shown in Section 4.

For the selection of parameters, first of all, the two sigma values were the variance of the kernel. The larger the variance, the greater the influence of the item on the weight (refer to the Gaussian filtering kernel function; the larger the sigma, the greater the degree of blurring, which, in fact, means that the spatial consistency of the weight has a more significant impact). In this paper, this method was used to filter invalid information in the mural. At the same time, as far as possible, we did not affect the image edge information of the mural. We consider that murals consist of many pigments and lines and that the extraction of refined sketches also requires relevant color information. In this paper, during the experiment, the neighborhood diameter d was set to 9. The standard deviation of the Gaussian function controlling grey scale similarity was set to 50. The standard deviation of the Gaussian function controlling spatial proximity was set to 75.

### 3.2. Edge Detection

In various types of images, different objects have distinct reflection characteristics to electromagnetic waves. This results in significant grey level changes at the intersection of objects and the background, thereby creating edges in the image. Edge detection technology utilizes grey-level change information to detect object edges, delineate object outlines, and achieve image segmentation. Edge detection is the first step in all boundary-based segmentation methods.

In this paper, we employ the Laplacian Edge method from the Kornia library. This method is compared against one traditional edge detection method and three deep learning-based methods.

#### 3.2.1. Laplacian Edge (Kornia)

The Laplacian operation is a linear combination of partial derivative operations and belongs to the second order differential operations. Compared with the traditional first-order differential operations, the boundaries obtained by the Laplacian operation are more detailed and contain more information. At first, a second-order differential discretization formula is defined. Then, an isotropic filter, named Laplacian operator based on this formula, is constructed with rotational invariance. The point where the second-order differentiation of the pixel value of the point is 0 is the edge point.

The 2D Laplace transform is based on the second order gradient and is defined as follows:

(3) $$\nabla^{2}f\left(x,y\right)=\frac{\partial^{2}f\left(x,y\right)}{\partial^{2}x}+\frac{\partial^{2}f\left(x,y\right)}{\partial^{2}y}\approx f(x+1,y)-f(x,y)-(f(x,y)-f(x-1,y))\;+f(x,y+1)-f(x,y)-(f(x,y)-f(x,y-1)\;\;\;\;\;\;\;\;\;\;\;\approx f(x+1,y)+f(x-1,y)+f(x,y+1)+f(x,y-1)-4f(x,y)$$

The convolution form is represented as the convolution of a matrix with a Laplace kernel. The original Laplace kernel and the transformation are shown in Equations (4) and (5):

(4) $$I=\left(\begin{array}{ccc} 0 & -1 & 0\\ -1 & 4 & -1\\ 0 & -1 & 0 \end{array}\right)$$

(5) $$\nabla^{2}f(x,y)=P*I$$

where the symbol ∗ represents the convolution operation; P is the image matrix; I is the Laplace kernel. 

**Figure 5:**
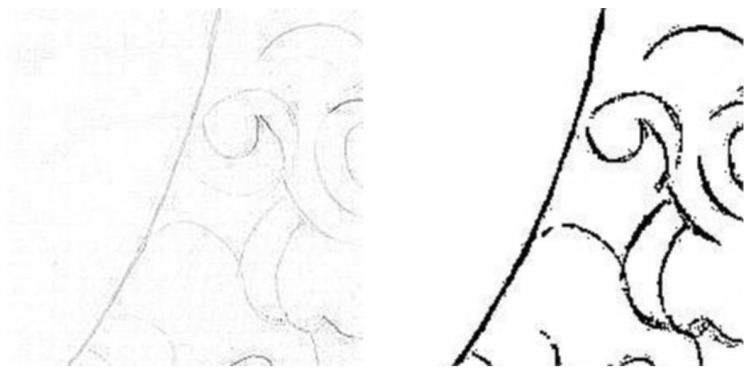
The left shows the detection results of the original Laplacian method, and the right shows the detection results of the Laplacian Edge used in this paper.

The above equation shows that the convolution of the image matrix P with the Laplace kernel I is essentially a calculation of the quadruple of the difference between the value at a position on the image and the average of its four neighboring directional points (horizontal, vertical). However, the original method is not good at recognizing lines in oblique directions in the image, as shown in Figure 5.

Traditional Laplacian methods detect result maps with fine grid-like strips, and the results are not well represented when detecting lines from oblique directions. The Laplacian method provided in Kornia [24] solves this problem. Kornia is an open-source computer vision library built on top of PyTorch that aims to help to better implement deep learning-oriented computer vision applications. Kornia provides classical and advanced image processing algorithms that can be embedded into neural networks and optimized during training. It provides high-level differentiable implementations of these algorithms. The Laplacian kernel is optimized to obtain the parameters as in Equation (6):

(6) $$I_{K}=\left(\begin{array}{ccc} 0.0625 & 0.0625 & 0.0625\\ 0.0625 & -0.5 & 0.0625\\ 0.0625 & 0.0625 & 0.0625 \end{array}\right)$$

As the algorithm is embedded in the neural network by Kornia, this Laplacian method excels in identifying line components in oblique directions and obtaining richer line information. This capability aligns well with the needs of refined sketch extraction for murals, differing subtly from the aims of traditional edge detection. We also used various edge detection methods, including advanced deep learning and traditional methods. Deep learning methods offer several advantages over traditional algorithms. They are fast, efficient, and often yield good results in conventional edge detection tasks. However, deep learning methods prioritize the integrity of edges, which often results in the loss of many refined line details. In this experiment, while a more complete sketch was obtainable by deep learning methods, the results often suffered from thick lines and loss of detail. This drawback makes these methods less suitable for extracting refined sketches of murals. 

#### 3.2.2. FNR

**Figure 6:**
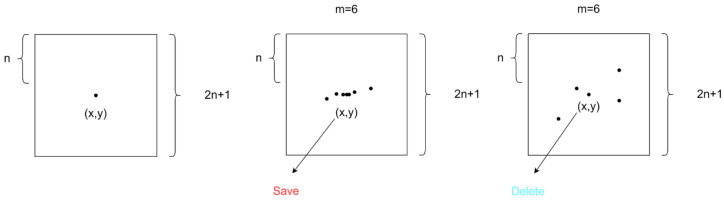
Schematic diagram of fine noise remover, where *n* and *m* are settable terms.

After the above steps to detect the sketch whilst it still existed as part of the fine noise, we designed a fine noise remover (FNR) to remove the possible noise in the mural while retaining the lines of the mural. The idea is to take the pixel point (*x*, *y*) as the center; the boundary length is 2*n* + 1 among the square pixel points, and each pixel point is judged on whether it is a black pixel point. If more than *m* points are black pixel points in the square area of the center point, the pixel point (*x*, *y*) is judged to be a part of the connecting line. Otherwise, it is regarded as an independent noise point. If the judgment result is independent noise, it is set to white in the image. As shown in Figure 6:

The remover passes each pixel point in the image to perform the operation to achieve the aim of noise removal while retaining the lines in the image. The parameters *n* and *m* can be adjusted according to the characteristics of the image. According to several experiments, *n* was set to 3 and *m* was set to 4 to reach the optimal effect in this work.

## 4. Experimental Results and Discussion

We used the simulated mural data to analyze the role of each step in the framework. The results for other experimental data will be shown in Section 4.2. All experiments were performed with 32 GB of RAM, CPU Inter(R) Xeon(R) Silver 4110 @ 2.10 GHz, and GPU NVIDIA Quadro P4000. All methods were implemented in Python 3.10, Porch 1.10.1, and Cuda 11.7.

### 4.1. Analysis of Each Step

#### 4.1.1. Preprocessing

The original digital image of the simulated mural was captured by a digital camera. The mural’s color information as rich, featuring lines often too similar to the background color. This similarity poses challenges for the subsequent detection methods used in sketch extraction. For instance, in the hair part of a character in the simulated mural, the thick black lines and the dark blue background color are hard to distinguish from the naked eye. This similarity can lead to the loss of line information when processed by subsequent algorithms. To address this, we employed color transformation as a data preprocessing method. This technique altered the color information of the painting area, highlighting the distinction between the lines and the background color.

#### 4.1.2. CLAHE

**Figure 7:**
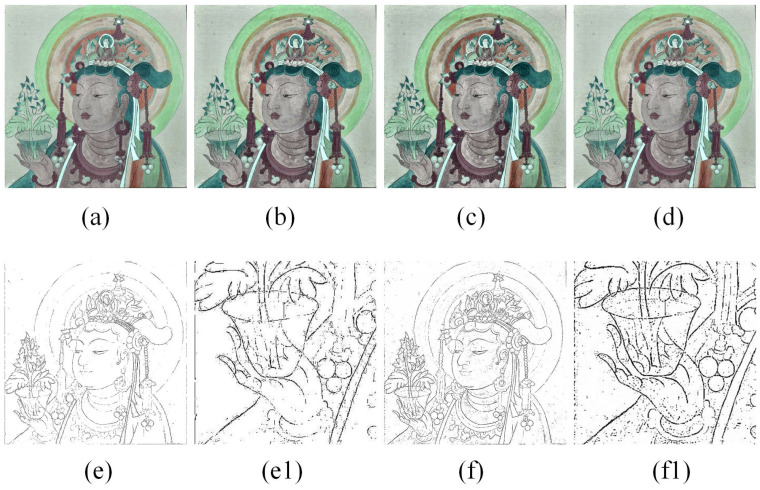
(**a**–**d**) Images enhanced by CLAHE. (**a**–**d**) The images obtained by setting the threshold of CLAHE to 1, 2, 3, and 4, respectively; (**e**) the result after directly extracting the original image, (**f**) the result after extracting the enhanced image, and (**e1**,**f1**) the corresponding local zoom-in details.

The mural contained lines, pigments, and areas where pigment had been repainted, making the boundaries between different-colored fields blurry. Consequently, the line information was not distinct enough. To improve the edge detection effect, we enhanced the image contrast using CLAHE, which also helped to suppress the noise generated during this process. For optimal results, we set the grid size to 8 × 8 and the threshold parameter to 2. As shown in Figure 7, setting the parameter to 3 or 4 resulted in over-enhancement, while a setting of 1 was insufficiently effective. Therefore, we selected a clip limit threshold value of 2. The enhanced contrast in the image is clearly evident. There are distinct differences between various color domains and between these domains and the lines. Additionally, the contrast between light color areas, the background lines, and the overall background is more pronounced. These distinctions are beneficial for the segmentation capability of the edge detector.

The experimental choice of using the CLAHE method for image enhancement was made to obtain as many lines and richer details as possible. Also, to verify the applicability of this step, the CLAHE-enhanced image and the original image were directly input into the edge detector, and the results are shown in Figure 7, illustrating that the enhanced image can portray more lines and details that match the actual image after edge detection, but inevitably produces noise.

#### 4.1.3. Bilateral Filtering

**Figure 8:**
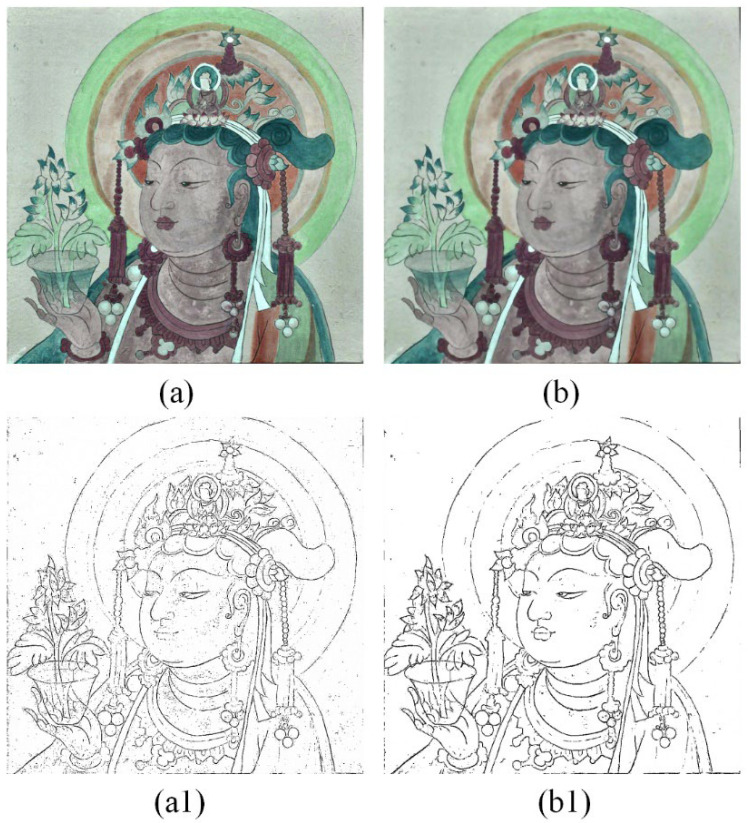
(**a**) The image after being enhanced by CLAHE, (**b**) the result of bilateral filtering of (**a**); (**a1**,**b1**) the corresponding sketch extraction results, respectively.

Preserving intact mural paintings is often challenging. Mural paintings usually have cluttered information. Additionally, the capturing of mural data is often interfered with by noise. While CLAHE effectively enhances image contrast to improve the extraction of mural painting sketches, it also amplifies this cluttered information to some extent. To obtain a more accurate sketch, it is crucial to address this cluttered information. At the same time, it must not affect the edge information of the mural paintings. The ordinary filter easily leads to the loss of edge information, so we introduced the bilateral filter to filter the invalid information in the image, which could preserve the integrity of the edge information. After CLAHE processing, bilateral filtering was applied to the image, as illustrated in Figure 8. As a result, the cluttered information, especially in the face region, was effectively faded and filtered. Simultaneously, the edge information was well-preserved and remained largely unaffected. Also, to verify this step’s applicability, the image enhanced by bilateral filtering was directly input into the edge detector. The results were compared with the original image’s detection results and the CLAHE-enhanced image’s detection results, respectively. As shown in Figure 8, bilateral filtering removes a significant amount of noise from the detection results while preserving all the edge information and increasing the quantity of valid information.

#### 4.1.4. Laplacian Edge (Kornia) with FNR

We employed the Laplacian Edge (Kornia) for image edge detection to obtain a refined mural sketch. The results shown in Figure 7 and Figure 8 are the edge maps obtained directly by using the Laplacian Edge (Kornia) method. It can be seen that although the result is good, there is still fine noise. We designed a fine noise remover to be added to the Laplacian Edge (Kornia). To maximally retain the accurate line information of the mural, the detection radius of the remover was set to 3, and the minimum number of points was set to 4. To confirm that these actions are required, we then applied the detection algorithm to both the simulated and enhanced murals and compared the accuracy with the real sketches of the simulated mural. The sketch extraction of the mural is slightly different from other edge detection targets, focusing more on predicting more lines and more complex lines to be extracted. Thus, we chose the Matthews correlation coefficient (MCC) as the statistical metric. The larger the value of MCC is, the better the performance. The expression of MCC is as follows:

(7) $$MCC=\frac{TP\times TN-FP\times FN}{\sqrt{\left(TP+FP)\times (TP+FN)\times (TN+FP)\times (TN+FN\right)}}$$

*TP* represents the number of true positives, *TN* represents the number of true negatives, *FP* represents the number of false positives, and *FN* represents the number of false negatives.

**Figure 9:**
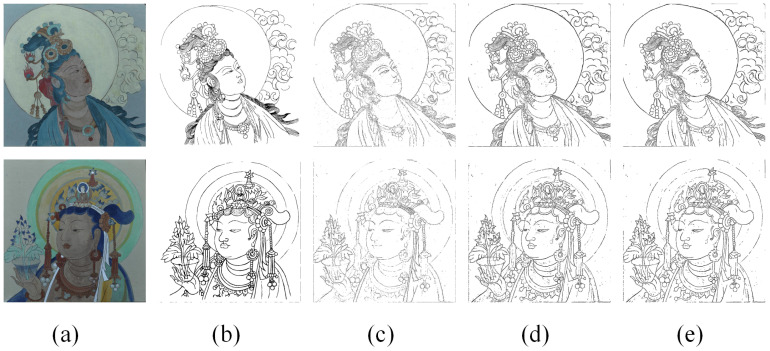
(**a**) The original painted cultural relics images; (**b**) ground truth; (**c**) the result after processing of the original image using the Laplacian Edge; (**d**) the result after processing of the enhanced image using the Laplacian Edge; (**e**) the result after processing of the enhanced image using the Laplacian Edge with FNR.

As shown in Figure 9 and Table 1, the results of the enhanced images show a significant improvement effect, both visually and in terms of accuracy.

**Figure 10:**
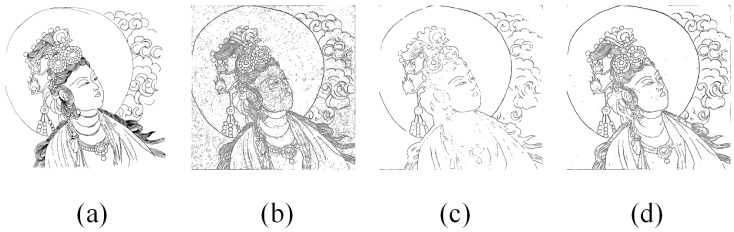
Ablation experiment: (**a**) ground truth; (**b**) the result of CLAHE-enhanced; (**c**) the result of Bilateral filtering-enhanced; (**d**) ours.

To demonstrate the applicability of our image enhancement techniques, we conducted ablation experiments. We selected CLAHE-enhanced and bilateral filtering-enhanced images of the ‘SHUIYUE’ as objects of study. We subjected the images to edge detection separately by the Laplacian Edge (Kornia) with FNR. The results of these experiments are presented in Figure 10 and Table 2. The results indicate that our proposed combination method achieves the highest accuracy and superior visual results, thus verifying the effectiveness of each enhancement step.

### 4.2. Comparison with Other Methods of Edge Detection

To obtain the optimal mural sketch, we conducted experiments on the enhanced image using a traditional edge detection method (Canny) and deep learning methods (HED, LDC [25], PiDiNet [26]). Canny is a traditional edge detection method, and the HED, LDC, and Pedant are deep learning methods. Obtaining an authentic sketch of the mural is challenging, making it difficult to produce a comprehensive mural sketch dataset. However, the edge information in mural images does not significantly differ from the edges in other data types. Therefore, we conducted experiments using pre-trained models provided by the network. We chose the structural similarity index (SSIM) as the statistical metric. The larger the value of SSIM is, the better the performance. The expression of SSIM is as follows:

(8) $$SSIM(x,y)=\frac{\left(2\mu_{x}\mu_{y}+c_{1})(\sigma_{xy}+c_{2}\right)}{\left(\mu_{x}^{2}+\mu_{y}^{2}+c_{1})(\sigma_{x}^{2}+\sigma_{y}^{2}+c_{2}\right)}$$

where $\mu_{x}$ and $\mu_{y}$ are the mean values of images *x* and *y*. $\sigma_{x}\;$and $\sigma_{y}$ are the standard deviation of *x* and *y*. $\sigma_{xy}$ is the covariance of image *x* and image *y*. $c_{1}$ and $c_{2}$ are constant.

**Figure 11:**
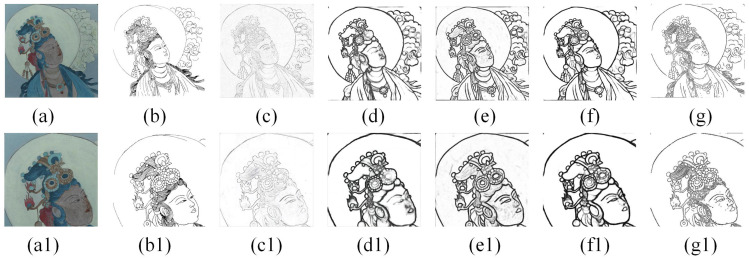
Comparison with other methods: (**a**) The original painted cultural relics images; (**b**) ground truth; (**c**) Canny; (**d**) HED; (**e**) LDC; (**f**) PiDiNet; (**g**) ours. The figures below (**a1**–**g1**) are the corresponding partially enlarged details.

The comparative results are shown in Figure 11 and Table 3. It is evident that even though both HED and LDC can obtain the complete edge better than our method, the refined and complex parts of the mural are not well recognized. Furthermore, the lines produced by the two deep learning techniques are somewhat thick, and only a few artifacts can be identified in the complex regions, with poor detection of the mural’s characters’ hair. PiDiNet only recognizes the outer contours well and does not obtain sketches of complex areas well. The mural lines are much finer than the edges of other objects and often need more refined and accurate sketches as evidence for future restoration. In this paper, the Laplacian Edge (Kornia) with FNR method shows better results in the refined extraction of mural sketches. It also has a good detection effect, particularly in extracting the complex parts of the character’s hair. Compared with other methods, our method can extract mural sketches with finer lines and more accurate information.

**Figure 12:**
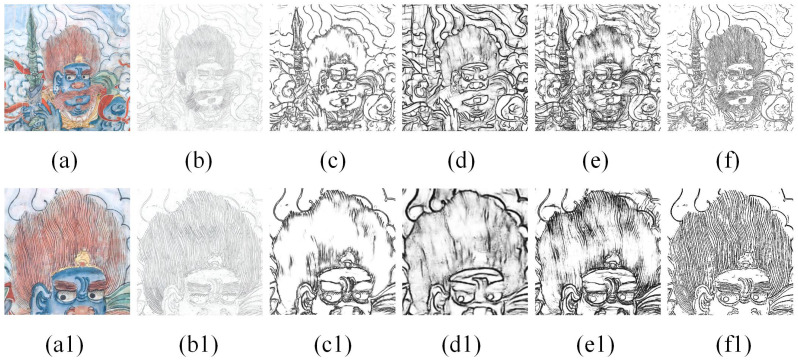
Comparison with other methods: (**a**) actual cultural relics images; (**b**) Canny; (**c**) HED; (**d**) LDC; (**e**) PiDiNet; (**f**) ours. The figures below (**a1**–**f1**) are the corresponding partially enlarged details.

In addition, to verify the applicability of our method, we used actual murals as study objects and conducted experiments using the above methods. The actual data are from a mural in Cave 5 West of the Yungang Grottoes. The comparative results are shown in Figure 12. We found that the results extracted from the actual mural were similar to the simulated mural experiments. The lines obtained by Canny were somewhat shallow and distorted. For example, the hair of a Buddha statue in a mural is drawn in one stroke, but two lines were detected. The lines obtained by HED and LDC were thick, and these methods do not identify lines in complex areas very well. As shown in Figure 12, we can find that hair regions are recognized as artifacts. PiDiNet has a nice effect, but the sketches obtained do not give a complete picture of what the mural really is. However, compared with these methods, our method is more suitable for extracting sketches of murals. The result of our method is relatively straightforward and almost free of artifacts, and the method can identify the texture features of the hair region well.

## 5. Conclusions

In this paper, we propose a novel method for extracting refined sketches from murals by utilizing image enhancement and edge detection techniques. This method uses image enhancement techniques to highlight the mural’s edge information while preventing noise, which is typically generated by over-enhancement. Moreover, the edge detection method proposed effectively extracts the complete outline of the mural and its refined texture features. According to our results, the method is highly efficient and produces objective outcomes. However, our method has some limitations. First, the objects to which our method applies are refined targets, such as murals. This method does not assure the accuracy of sketches obtained from severely deteriorated or damaged murals. And although the results extracted by our method are good, some noise is still apparent. At the same time, how to evaluate the experimental results of murals without ground truth is a problem that needs to be solved. We think it would be a good research direction to produce a large dataset of sketches of murals. In the future, we will continue to investigate these issues to improve the accuracy of sketch extraction.

## Figures and Tables

**Table 1:** Comparison of the accuracy of the extraction results between the enhanced images and the original image.

Subjects	MCC
DAFO (Original)	0.0685
DAFO (Enhanced)	0.0842
SHUIYUE (Original)	0.0811
SHUIYUE (Enhanced)	0.0972

**Table 2:** Comparison of the precision of the extracted results.

Method	MCC
CLAHE-enhanced	0.0922
Bilateral filtering-enhanced	0.0498
Ours	0.0972

**Table 3:** Comparison of accuracy with other edge detection methods.

Method	SSIM
Canny	0.1997
HED	0.4981
LDC	0.4623
PiDiNet	0.5062
Ours	0.5615

## Data Availability

Data is unavailable due to privacy restrictions. We still need these simulated mural data and real mural data for our subsequent experiments.
